# Safety of artemether-lumefantrine exposure in first trimester of pregnancy: an observational cohort

**DOI:** 10.1186/1475-2875-13-197

**Published:** 2014-05-27

**Authors:** Dominic Mosha, Festo Mazuguni, Sigilbert Mrema, Esperanca Sevene, Salim Abdulla, Blaise Genton

**Affiliations:** 1 Ifakara Health Institute, Rufiji HDSS, P.O Box 40, Rufiji, Tanzania; 2 Swiss Tropical and Public Health Institute, University of Basel, Basel, Switzerland; 3 Universidade Eduardo Mondlane, Faculdade de Medicina, Manhica Health Research Centre, Maputo, Mozambique; 4 Department of Ambulatory Care and Community Medicine & Division of Infectious Diseases, University Hospital, Lausanne, Switzerland

**Keywords:** Pregnancy, Safety, Artemether-lumefantrine, Exposure

## Abstract

**Background:**

There is limited data available regarding safety profile of artemisinins in early pregnancy. They are, therefore, not recommended by WHO as a first-line treatment for malaria in first trimester due to associated embryo-foetal toxicity in animal studies. The study assessed birth outcome among pregnant women inadvertently exposed to artemether-lumefantrine (AL) during first trimester in comparison to those of women exposed to other anti-malarial drugs or no drug at all during the same period of pregnancy.

**Methods:**

Pregnant women with gestational age <20 weeks were recruited from Maternal Health clinics or from monthly house visits (demographic surveillance), and followed prospectively until delivery.

**Results:**

2167 pregnant women were recruited and 1783 (82.3%) completed the study until delivery. 319 (17.9%) used anti-malarials in first trimester, of whom 172 (53.9%) used (AL), 78 (24.4%) quinine, 66 (20.7%) sulphadoxine-pyrimethamine (SP) and 11 (3.4%) amodiaquine. Quinine exposure in first trimester was associated with an increased risk of miscarriage/stillbirth (OR 2.5; 1.3–5.1) and premature birth (OR 2.6; 1.3–5.3) as opposed to AL with (OR 1.4; 0.8–2.5) for miscarriage/stillbirth and (OR 0.9; 0.5–1.8) for preterm birth. Congenital anomalies were identified in 4 exposure groups namely AL only (1/164[0.6%]), quinine only (1/70[1.4%]), SP (2/66[3.0%]), and non-anti-malarial exposure group (19/1464[1.3%]).

**Conclusion:**

Exposure to AL in first trimester was more common than to any other anti-malarial drugs. Quinine exposure was associated with adverse pregnancy outcomes which was not the case following other anti-malarial intake. Since AL and quinine were used according to their availability rather than to disease severity, it is likely that the effect observed was related to the drug and not to the disease itself. Even with this caveat, a change of policy from quinine to AL for the treatment of uncomplicated malaria during the whole pregnancy period could be already envisaged.

## Background

Over 60% of all pregnancies globally are at risk of malaria and more than 32 million are in sub-Sahara Africa [1]. Malaria infection is associated with high maternal and perinatal mortality in tropical and subtropical regions [2]. Severe maternal anaemia, intrauterine growth retardation, intrauterine death, stillbirth, premature delivery and low birth-weight are some of the reported substantial direct risks of malaria in pregnancy [2,3]. Although malaria in pregnancy is a serious public health problem, there is limited information available regarding safety profile of most of licensed anti-malarial in pregnancy because pregnant women are routinely not involved in clinical trials related to drug development for fear of harming the women and or developing foetus [4].

Artemisinin-based combination therapy (ACT) is the most effective drug combination for *Plasmodium falciparum* malaria and has been recommended by the World Health Organization (WHO) as a treatment of choice for the treatment of *P. falciparum* malaria [5]. ACT is only recommended in pregnancy during second and third trimester, but not in first trimester, unless they are the only treatment available, or if the patient’s life is threatened. Safety concerns of artemisinins in first trimester are the associated risks of visceral and skeletal anomalies following animal studies in early stage of pregnancy [6,7]. Two previous small-scale studies assessing Zambian and Sudanese pregnant women exposed to artemisinin during first trimester could not find any association between drug exposure and maternal or birth adverse outcomes [8,9]. However, evidence is still scarce to ensure safety of ACT during first trimester.

Artemether-lumefantrine (AL) (20 mg and 120 mg, respectively) (Coartem©, Novartis Pharm AG) is one of the most popular and efficacious fixed dose of ACT which is currently available [10]. AL was introduced in Tanzania as a first-line therapy for malaria in 2006 to replace sulphadoxine-pyrimethamine (SP) [11]. Inadvertent exposure to artemisinin during first trimester of pregnancy is possible due to its high availability at a subsidized cost in both private and public health facilities in the country [12,13]. Furthermore, self-treatment of malaria without consulting a trained professional is common in sub-Sahara Africa; indeed, 70% of malaria episodes in rural Africa and 50% in urban areas are self-treated cases [14]. It is, therefore, important to take advantage of the latter to extend the margin safety information of artemisinin compounds in pregnancy by evaluating maternal and birth outcomes of inadvertently AL exposure to women in their first trimester.

There is increasing evidence supporting efficacy, safety and tolerability of ACT, which outweigh the advantages of quinine in treating malaria [15,16]. Despite its reactogenicity profile and several reports of resistant strains of *P falciparum*[17,18], quinine remains the only recommended drug for treating both uncomplicated and complicated *P falciparum* malaria during first trimester of pregnancy [5,11]. The present study aims at assessing the maternal and birth outcomes in pregnant women who were inadvertently exposed to AL during first trimester in comparison to those of women exposed to other anti-malarial drugs or no drug at all during the same period of pregnancy using two Health Demographic Surveillance System (HDSS) platforms in Tanzania.

## Methods

### Study area

The study was conducted in Rufiji and Kigoma HDSS in Tanzania. Rufiji HDSS is in a rural setting while Kigoma HDSS is in an urban one, both areas have moderate to high malaria transmission intensity [19]. The study involved a total of 22 health facilities in the two HDSS sites. There was no clinical interventional research activity in the area during the study period.

### Study design

The study enrolled pregnant women with gestational age of 20 weeks and below between April 2012 and March 2013. Only women residing in HDSS were eligible for the study. They were recruited from Reproductive and Child Health (RCH) clinic during their routine visits and from the community through monthly round-based house visits. The set-up of HDSS allows identification of pregnancy status in women of childbearing age through routine HDSS quarterly census. On the day of enrolment, participants were interviewed for obstetrics and previous medical history including history of chronic illness or disease, use of alcohol and smoking. Important laboratory test such as maternal haemoglobin level, screening for HIV and syphilis were performed. Use of any anti-malarial during first trimester of the presenting pregnancy was the key question during interview. Information regarding the reported drug used by a participant was verified by assessing patient’s medical log in the attended health facility, prescription sheet and maternal RCH card. Assessment of RCH card and patient’s medical log were also carried out for participants who reported no drug intake. In case of discordance between the facility medical log and what the participant had reported regarding the used medicine, participant’s information was considered the truth after further interview to verify specifications of the said medicine, whether it was used or not. Participants who had inadvertently used AL for malaria treatment in first trimester were compared with pregnant women who were treated with either quinine (Qn), sulphadoxine-pyrimethamine (SP), amodiaquine or women who had not used anti-malarial drug(s) at all during the same period of pregnancy. Thus, women were not randomized but grouped to the study arm according to their anti-malarial exposure history in first trimester.

Women were followed on monthly basis until delivery to monitor pregnancy and birth outcomes. The assessed pregnancy outcome included maternal mortality, spontaneous abortion (pregnancy lose ≤ 28 weeks of gestation), ectopic gestation, stillbirth and live birth. Birth outcome included birth weight, maturity status at birth [estimated from the last normal menstrual period (LNMP), or fundal height examination, when the LNMP was unknown] and presence of congenital anomalies. All newborns were assessed for congenital abnormalities post-delivery by a study clinician or health facility midwife. Screening for congenital abnormalities was performed under the guidance of a specific developed checklist. The screening was limited to identify external abnormalities regardless of the degree of severity. No examination was performed to determine neurological score for sensory or motor patterns.

### Primary endpoint

Primary endpoints of the study were pregnancy and baby outcomes. Pregnancy outcome included miscarriage, stillbirth or live birth whereas baby outcome included birth weight and prematurity status at birth. Stillbirth was defined as a baby born with no signs of life at or after 28 weeks of gestation. Low birth weight was defined as a birth weight below 2,500 grams and premature was defined as birth before 37 weeks of gestational age.

### Statistical analysis

STATA® 12.0 (Stata Corporation, College Station, Texas, USA) was used for data analysis. Numerical variables were summarized into mean and standard deviation. Categorical variables were summarized using cross tabulation to estimate different proportion. The effect of demographic and pregnancy characteristics on primary endpoint of the study was assessed by bivariate analysis. Explanatory variables were included in the multivariate analysis if the variable had p-value < 0.2 in bivariate analysis. Logistic regression model were used to estimate the odds ratio (OR) for the associated between binary pregnancy outcomes (birth outcome, birth weight and birth maturity status) and medicine exposure. Two sided Wald test P-values are presented.

### Ethics

Ethical approval was granted by the Ifakara Health Institute (IHI) ethical review board and the National Institute for Medical Research (NIMR) ethical committee. Written informed consent was obtained from all participants.

## Results

**Figure 1:**
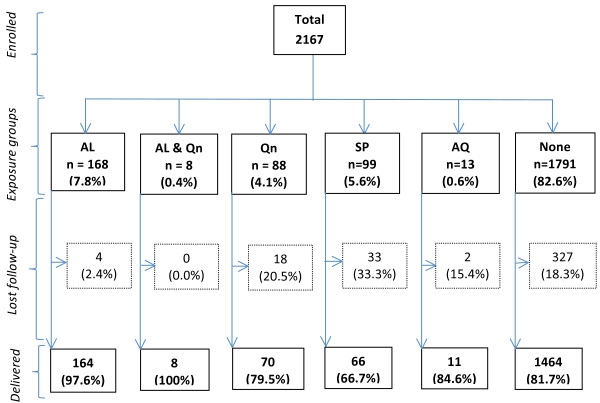
**Flow chart of participants in the study.** AL = Artemether-lumefantrine; Qn = Quinine; SP = Sulphadoxine-pyrimethamine; AQ = Amodiaquine; None = No anti-malarial.

A total of 2,167 pregnant women were enrolled in the study and 1,783 (82.3%) were followed until delivery (Figure 1). 19.2% (342) were recruited from the community through house visit and 80.8% (1441) from the facility during their routine RCH clinic visits. 602 (33.8%) women were recruited in first trimester of pregnancy with mean gestational age of 10.5 [standard deviation (SD) 2.6] and 1181 (66.2%) during the first half of second trimester of pregnancy with mean gestation age of 16.9 (1.5). 559 (31.4%) were primigavidae, 336 (18.8%) secundigravidae and 888 (49.8%) were multigravidae with gravidity of 3 and above. Important demographic and clinical characteristics are summarized in Table 1.

**Table 1:** Characteristics of pregnant women enrolled in the study

**Characteristics**	**First trimester**	**First half of second trimester**	**All trimesters**
	**n = 602**	**n = 1181**	**n = 1783**
Mean age, (years)*	25.7 (6.8; 14 – 49)	25.9 (6.7; 13 – 46)	25.8 (6.8; 13 – 49)
Mean BMI*	23.2 (3.9; 14.2 – 39.6)	23.3 (3.6; 14.0 – 42.5)	23.4 (3.7; 14.0 – 42.5)
Mean gestational age, (weeks)*	10.5 (2.6; 3 – 12)	16.9 (1.5; 13 – 20)	14.8 (3.7; 3 – 20)
Gravidity^#^			
Primigravidae	182 (30.0)	377 (31.9)	559 (31.4)
Secundigravidae	109 (18.0)	227 (19.2)	336 (18.8)
3 – 4 pregnancies	180 (29.6)	315 (26.7)	495 (27.8)
≥ 5 pregnancies	131 (21.4)	262 (22.2)	393 (22.0)
Recruited sites^#^			
Health facility	468 (22.3)	973 (82.4)	1441 (80.8)
Home	134 (77.7)	208 (17.6)	342 (19.2)
Drinking alcohol^#^	14 (2.7)	27 (2.3)	41 (2.3)
Smoking cigarette^#^	5 (0.8)	2 (0.2)	7 (0.4)
Haemoglobin level (g/dl)*	10.8 (1.5; 5.0 – 14.6)	10.7 (1.5; 5.4 – 14.9)	10.7 (1.5; 5.0 – 14.9)
HIV status^#^			
Negative	533 (88.5)	1086 (92.0)	1619 (90.8)
Positive	18 (3.0)	46 (3.9)	64 (3.6)
No results	51 (8.5)	49 (4.1)	100 (5.6)
Syphilis test^#^			
Negative	521 (86.5)	1082 (91.6)	1603 (89.9)
Positive	12 (2.0)	15 (1.3)	27 (1.5)
No results	69 (11.5)	84 (7.1)	153 (8.6)

*Abbreviation*: BMI = Body Mass Index.

*Represents data presented in mean, (standard deviation [SD]; range).

^#^Represent data presented in number (%).

### Drug exposure

319 (17.9%) women used anti-malarial in first trimester of pregnancy because of a morbid episode. 164 (51.4%) used AL only, 70 (21.9%) quinine only, 8 (2.5%) both AL and quinine, 66 (20.7%) SP and 11 (3.4%) amodiaquine. At least 88% of study women used three group of drugs that are in antenatal intervention as recommended by the Ministry of Health [11] namely, anthelminthic, sulphadoxine-pyrimethamine (SP) for intermittent preventive treatment of malaria (IPTp) and iron and folic acid supplementation. Anthelminthic and IPTp-SP are prescribed in second and third trimester of pregnancy. 1579 (88.6%) used anthelminthic (mebendazole), 1626 (91.2%) used iron and folic acid supplementation and 1636 (91.8%) used at least one dose of IPTp-SP.

### Pregnancy outcome and anti-malarial exposure

Among 1783 deliveries, there were 5 maternal deaths that occurred within 24 hours, three were due to post-partum haemorrhage and the remaining two each was secondary to eclampsia and disseminated intravascular coagulopathy (DIC), respectively. Pregnancy outcomes included 44 (2.5%) abortions, 62 (3.5%) stillbirth and 1677 (94.1%) live births. Baby outcomes included 81 (4.8%) low birth weight babies and 113 (6.7%) premature births. 23 (1.3%) of the newborns were identified to have congenital anomalies at birth including, polydactyl 17 (73.9%), club foot 2 (8.7%), genital defect 2 (8.7%), spina bifida 1 (4.3%) and cardiac defect 1 (4.3%). Congenital anomalies were identified in four exposed groups namely AL only (1 [0.6%] of 164), quinine only (1 [1.4%] of 70), SP (2 [3.0%] of 66), and non-anti-malarial exposed group (19 of [1.3%] of 1464). The median gestational age of anti-malarial exposure in the four exposure groups with congenital anomalies was 9 (6 – 10) weeks. Table 2 summarizes pregnancy outcomes parameters in relation to anti-malarial exposure status in first trimester.

**Table 2:** Pregnancy and baby outcomes in relation to anti-malarial exposure status during first trimester

**Pregnancy outcome**	**AL only**	**AL & Quinine**	**Quinine only**	**SP**	**Amodiaquine**	**None**
	**164 (%)**	**8 (%)**	**70 (%)**	**66 (%)**	**11 (%)**	**1464 (%)**
Abortion	5 (3.0)	2 (25.0)	3 (4.3)	0 (0.0)	0 (0.0)	34 (2.3)
Stillbirth	6 (3.7)	0 (0.0)	5 (7.1)	2 (3.0)	0 (0.0)	49 (3.3)
Live birth	153 (93.3)	6 (75.0)	62 (88.6)	64 (97.0)	11 (100)	1381 (94.3)
Birth maturity*						
Preterm birth	8 (5.2)	2 (33.3)	8 (12.9)	7 (10.9)	0 (0.0)	88 (6.4)
Full term birth	145 (94.8)	4 (66.7)	54 (87.1)	57 (89.1)	11 (100)	1293 (93.6)
Birth weight*						
Low birth weight	8 (5.2)	1 (16.7)	1 (1.6)	2 (3.1)	0 (0.0)	69 (5.0)
Normal birth weight	145 (94.8)	5 (83.3)	61 (98.4)	62 (96.9)	11 (100)	1312 (95.0)
Congenital anomalies	1 (0.6)	0 (0.0)	1 (1.4)	2 (3.0)	0 (0.0)	19 (1.3)

Abbreviation: AL = Artemether-lumefantrine; SP = Sulphadoxine-pyrimethamine.

*Excluded abortion and stillbirth outcomes.

Quinine exposure during first trimester was associated with an increased risk of miscarriage/stillbirth (adjusted OR 2.5; 95%CI 1.3 – 5.1; p = 0.009) and premature birth (adjusted OR 2.6; 95%CI 1.3 – 5.3; p = 0.007) as opposed to AL, SP and amodiaquine exposure which were not associated with increased risk of either miscarriage/stillbirth, low birth weight or premature birth [see details in Table 3].

**Table 3:** Pregnancy outcomes in relation to anti-malarial exposure status in first trimester

**Variables**	**Outcomes**		**Crude OR**	**P**^**μ**^	**Adjusted OR**^**α**^	**P**^**μ**^
			**(95% CI)**		**(95%CI)**	
**Birth outcome**	**MC/SB**	**Live birth**				
	**n (%)**	**n (%)**				
AL exposure						
Yes	13 (12.3)	159 (9.5)	1.3 (0.7 – 2.4)	0.348	1.4 (0.8 – 2.5)	0.295
No	93 (87.7)	1518 (90.5)				
Quinine exposure						
Yes	10 (9.4)	68 (4.1)	2.5 (1.2 – 4.9)	0.011	2.5 (1.3 – 5.1)	0.009
No	96 (90.6)	1609 (95.9)				
SP exposure						
Yes	2 (1.9)	64 (3.8)	0.5 (0.1 – 2.0)	0.318	0.5 (0.1 – 2.0)	0.312
No	104 (98.1)	1613 (96.2)				
Amodiaquine exposure						
Yes	0 (0.0)	11 (0.7)	- (0)	-	- (0)	-
No	106 (100)	1666 (99.3)				
No anti-malarial exposure						
Yes	83 (78.3)	1380 (82.3)	0.8 (0.5 – 1.3)	0.301	0.8 (0.5 – 1.2)	0.260
No	23 (21.7)	297 (17.7)				
**Birth weight (grams)**	**< 2500**	**≥ 2500**				
	**n (%)**	**n (%)**				
AL exposure						
Yes	9 (11.1)	150 (9.4)	1.2 (0.6 – 2.5)	0.608	1.2 (0.6 – 2.5)	0.573
No	72 (88.9)	1446 (90.6)				
Quinine exposure						
Yes	2 (2.5)	66 (4.1)	0.6 (0.1 – 2.4)	0.463	0.6 (0.1 – 2.4)	0.461
No	79 (97.5)	1530 (95.9)				
SP exposure						
Yes	2 (2.5)	62 (3.9)	0.6 (0.2 – 2.6)	0.520	0.7 (0.2 – 3.0)	0.639
No	79 (97.5)	1534 (96.1)				
Amodiaquine exposure						
Yes	0 (0.0)	11 (0.7)	- (0)	-	- (0)	-
No	100 (100)	1585 (99.3)				
No anti-malarial exposure						
Yes	69 (85.2)	1311 (82.1)	1.3 (0.7 – 2.3)	0.485	1.2 (0.6 – 2.3)	0.564
No	12 (14.8)	285 (17.9)				
**Maturity status at birth**	**Preterm**	**Term**				
	**n (%)**	**n (%)**				
AL exposure						
Yes	10 (8.9)	149 (9.5)	0.9 (0.5 – 1.8)	0.812	0.9 (0.5 – 1.8)	0.865
No	103 (91.1)	1415 (90.5)				
Quinine exposure						
Yes	10 (8.9)	58 (3.7)	2.5 (1.3 – 5.1)	0.010	2.6 (1.3 – 5.3)	0.007
No	103 (91.1)	1506 (96.3)				
SP exposure						
Yes	7 (6.2)	57 (3.6)	1.7 (0.8 – 3.9)	0.177	1.8 (0.8 – 4.1)	0.160
No	106 (93.8)	1507 (96.4)				
Amodiaquine exposure						
Yes	0 (0.0)	11 (0.7)	- (0)	-	- (0)	-
No	113 (100)	1553 (99.3)				
No anti-malarial exposure						
Yes	88 (77.9)	1292 (82.6)	0.7 (0.5 – 1.2)	0.205	0.7 (0.5 – 1.1)	0.168
No	25 (22.1)	272 (17.4)				

*Abbreviations*: AL = Artemether-lumefantrine; MC = Miscarriage; SB = Stillbirth; SP = Sulphadoxine-pyrimethamine.

RR = Relative Risk; CI = Confidence Interval.

^**μ**^Estimated from the logistic regression model with Wald type P-value.

^**α**^Adjusted for age and parity.

Maternal age and parity were assessed to determine their effect on pregnancy outcome as potential confounders of the drug effect. Increase of maternal age in years was associated with 5% decreased risk of low birth weight (OR 0.95; p = 0.009), 5% increased risk of miscarriage/stillbirth (OR 1.05; p = 0.001), and 3% increased risk of preterm birth (OR 1.03; p = 0.016). Multigravidae had 50% decreased risk of low birth weight (OR 0.5; p = 0.006), 60% increased risk of miscarriage/stillbirth (OR 1.6; p = 0.048), and 30% increased risk of preterm birth (OR 1.3; p = 0.099) compared to primigravidae.

## Discussion

The study findings provide further evidence on the safety profile of AL use in early pregnancy to treat malaria. It differs from previous first trimester artemisinins derivatives safety studies [8,9] by having a larger sample size and a broader comparative exposure group. Also, the low mean gestational age at enrolment improves accuracy of drug exposure history, and thus reduces recall bias. It also increases the chances of identifying adverse pregnancy outcomes which commonly occurs during early stage of pregnancy, such as abortion [20].

Although AL is not recommended as first-line treatment for malaria during first trimester of pregnancy, it was used by 54% of women in this indication. Exposure to AL in first trimester was twofold higher than quinine, the drug of choice for malaria treatment during first trimester in Tanzania [11]. This observation suggests that AL is a popular drug. It reflects its high accessibility in most of the health facilities and by drug vendors in the country [12,13]. In practice, quinine was frequently out-of-stock and its replacement could easily take several weeks, particularly in public health facilities. The latter may explain why the efforts of study team to remind clinicians in study health facilities about contraindication of AL in first trimester had little effect. Since shortage of drugs is common in resource-limited settings [21,22], inadvertent or voluntary exposure to contraindicated drugs is inevitable. Limited access to quinine may also explain the observed high SP and amodiaquine exposure, drugs which are currently not recommended for treating malarial illness [5].

Quinine exposure was associated with a two-fold increased risk of miscarriage, stillbirth and preterm birth. The harmful effect of quinine during pregnancy has been known for a long time. Its abortive properties in relation to the induction of uterine contractions have long been reported by Maxwell [23]. The strength and prolongation of these contractions were reported to be dose dependent. A randomized control trial in Uganda showed oral quinine to have a two-fold increased incidence of adverse effects compared to AL among pregnant women treated for uncomplicated malaria in second and third trimesters. There were nearly two-fold increases in intrauterine foetal deaths in the quinine group than in the AL one, although the numbers were low. On the other hand, there was no difference in proportions of spontaneous abortions in the two study groups [16].

In the present study, these adverse pregnancy outcomes were not observed following AL, SP or amodiaquine exposure, which suggests that the deleterious effect of quinine was more related to the drug itself, rather than to the malaria episode. This is supported by the observation made by fieldworkers that quinine was not given to a particular group of women because of more severe disease, but just because AL was more readily accessible on the shelf of the health facility. Also, all women took quinine tablets, and not intravenous doses, which suggest a similar degree of severity of the disease in women who took quinine and AL. However, since it was not a proper randomized double-blind controlled trial, it is not possible to formally exclude a selection bias that would lead to different effects of the malaria disease itself. Whatever is the cause, the magnitude of the adverse effects associated with quinine exposure is alarming, when considering that this drug is viewed at present as the safest anti-malarial drug in first trimester. There is a remote possibility of a deleterious effect of AL on the foetus, and hence on infant development, that could not be assessed at this stage in the study. We hope to be able to definitely exclude an adverse consequence of AL exposure during pregnancy on the infant when analysing the results of the 12-month follow-up of the offsprings. Precise information on neurological scores, including motor and sensory patterns, should assist policy decisions after careful analysis of the time of anti-malarial exposure. The preliminary results of the first infant cohort are encouraging.

The observed prevalence of 1.3% congenital anomaly in the present study is lower than the global prevalence (3.0%) estimated by WHO [24]. No national figures of congenital anomalies are available for comparison in Tanzania. There are obvious limitations to screen for external anomalies only during the neonatal period: This may lead to an underestimation of the true prevalence of congenital abnormalities, which may appear later in life. Congenital anomalies was twice in the non-anti-malarial exposed group compared to AL exposed group (1.3% vs 0.6%). Polydactyly was the most reported congenital anomaly (74%), but it is believed to be genetically determined rather than triggered by external exposure [25]. In animal studies, umbilical hernia has been reported to be associated with artemisinin exposure during pregnancy [6]. The present study had limitation to assess occurrence of umbilical hernia since the newborns were screened only once at the time of delivery. At this time, hernias may hardly present, and cannot, therefore, be identified. Also, umbilical hernia is commonly observed in most parts of Africa and is not viewed as an abnormality, it is often not brought to medical attention unless it manifests itself with complications such as intestinal obstruction [26,27].

## Conclusion

Exposure to AL in first trimester was more common than to any other anti-malarial drugs. Quinine exposure was associated with adverse pregnancy outcome, which was not the case for other anti-malarials. Since AL and quinine were used according to their availability rather than to disease severity, it is likely that the effect observed was related to the drug, and not to the disease itself. More information of developmental milestone up to 12 months is needed to rule out any adverse effect on infancy as a result of AL exposure in first trimester. Even with this caveat, a change of policy from quinine to AL for the treatment of uncomplicated malaria during the whole pregnancy period could be already envisaged.

## Competing interests

BG has received in the past a research grant from Novartis Pharma to work on the effect of artemether-lumefantrine introduction on child mortality and malaria transmission in Tanzania. Novartis Pharma had no involvement in the present project.

## Authors’ contributions

The study was designed by DM and BG, assisted by ES and SA. Enrolment and follow-up of participants in the field was coordinated by DM and SM. FM was responsible for data entry and analysis. DM and BG wrote the first draft of the manuscript. All authors participated in data interpretation, review and approved the final manuscript.
